# Change of water consumption and its potential influential factors in Shanghai: A cross-sectional study

**DOI:** 10.1186/1471-2458-12-450

**Published:** 2012-06-18

**Authors:** Hanyi Chen, Yaying Zhang, Linlin Ma, Fangmin Liu, Weiwei Zheng, Qinfeng Shen, Hongmei Zhang, Xiao Wei, Dajun Tian, Gengsheng He, Weidong Qu

**Affiliations:** 1 Key Laboratory of Public Health Safety, Ministry of Education, Department of Environmental Health, School of Public Health, Fudan University, Shanghai, 200032, China; 2 Center for Disease Control and Prevention of Yangpu district, Shanghai, China; 3 Department Nutrition and Food Hygiene, School of Public Health, Fudan University, Shanghai, 200032, China

## Abstract

**Background:**

Different water choices affect access to drinking water with different quality. Previous studies suggested social-economic status may affect the choice of domestic drinking water. The aim of this study is to investigate whether recent social economic changes in China affect residents’ drinking water choices.

**Methods:**

We conducted a cross-sectional survey to investigate residents’ water consumption behaviour in 2011. Gender, age, education, personal income, housing condition, risk perception and personal preference of a certain type of water were selected as potential influential factors. Univariate and backward stepwise logistic regression analyses were performed to analyse the relation between these factors and different drinking water choices. Basic information was compared with that of a historical survey in the same place in 2001. Self-reported drinking-water-related diarrhoea was found correlated with different water choices and water hygiene treatment using chi-square test.

**Results:**

The percentage of tap water consumption remained relatively stable and a preferred choice, with 58.99% in 2001 and 58.25% in 2011. The percentage of bottled/barrelled water consumption was 36.86% in 2001 and decreased to 25.75% in 2011. That of household filtrated water was 4.15% in 2001 and increased to 16.00% in 2011. Logistic regression model showed strong correlation between one’s health belief and drinking water choices (*P* < 0.001). Age, personal income, education, housing condition, risk perception also played important roles (*P* < 0.05) in the models. Drinking-water-related diarrhoea was found in all types of water and improper water hygiene behaviours still existed among residents.

**Conclusions:**

Personal health belief, housing condition, age, personal income, education, taste and if worm ever founded in tap water affected domestic drinking water choices in Shanghai.

## Background

The quality of drinking water is critical for public health, with estimation of 80% of diseases in developing country, 4.0% global deaths and 5.7% the total disease burden (in DALYs) worldwide caused by poor water [1,2]. Various drinking water choices affect access to quality of water and then induce different risk for health [3,4]. Studies have suggested drinking water choices may vary by gender, age, education, economic status, risk perception and personal health belief [5-11]. As these factors are sociocultural, regional and temporal diversity could affect drinking water choices [9,12]

Previous investigations regarding influential factors of drinking water choices were widely conducted in developed countries [5-11]. However, there are few studies carried out in China, where significant social and economic achievements have taken place in the past three decades [13]. Indeed, benefited from a comprehensive development, lifestyle and health beliefs have extremely changed in recent years. Access to safe drinking water has become the first health claim and major goal for public and government in China [14,15]. Since the late 1980’s, drinking water choices have become increasingly diverse. Bottled water, barrelled water, household filtrated water successively entered into family and became the popular alternate of tap water for residents. Understanding the influential factors of domestic drinking water choices is important for health care providers, drinking water suppliers, and public health decision makers to ensure drinking water quality and guide the public to drink in a proper way [16,17].

Based on these facts, a cross-sectional survey was conducted in Yangpu district of Shanghai in summer 2011, where centralized filtrated water was first supplied directly to community residents in China and the historical survey was done in 2001 [18]. The possible influential factors including gender, age, education, annual income, housing condition, risk perception and personal health belief were investigated. In addition, self-reported drinking-water-related diarrhoea and daily drinking water hygiene were also investigated to provide a comprehensive analysis regarding different drinking water choices.

Our study is intended (1) to investigate change in Shanghai residents’ drinking water choices under the background of market economy; (2) to explore influential factors on domestic drinking water choices in China today; (3) to find out current misunderstandings in consumption of different water.

## Methods

A cross-sectional face-to-face survey of 416 respondents in Yangpu district was conducted in 2011. Sample size was calculated to achieve 90% power using an α of 0.05. At least 380 samples should be taken in order to tell if a difference of the interested parameter exists. Given potential loss of samples, an extra 10% was added, making the final sample size to be 416. Residents living in Yangpu district for more than 1 year were randomly selected by their age, gender and housing condition. Children above 7 were also included as they were believed to be able to express themselves clearly.

The study was approved by the ethical committee of Fudan University. Written informed consent was obtained from every respondent prior to participation. Questionnaires were performed by rigorously trained graduate students and health clinicians in local Centre for Disease Control & Prevention (CDC) and community health care. The final sample size is 400 after excluding incomplete or missing data

### Questionnaire development and study factors selection

The questionnaire was first designed and evaluated by four epidemiologists. A pilot study of 2% sample size was then carried out to verify its feasibility and to confirm if the factors included were representative of influential factors reflected by the residents. Ambiguous questions were then revised and sequence of the questionnaire was re-modified by the epidemiologists. The influential factors were finally decided to be (1) social demographic factors as gender, age, education, annual income and housing condition; (2) risk perception (personal subjective sensory perception for tap water); (3) personal belief in what type of water they considered cleanest or safest. The questionnaire was translated into English and provided as Additional file 1.

Specific age was asked but later divided into four groups in analysis-juvenile group (<18 years), young group (18–34 years), middle-aged group (35–59 years) and elder group (≥60 years), referring to the age classification in Shanghai Statistical Almanac [19].

Annual income was categorized based on three points: the lowest wage standard in 2010 Shanghai, a Shanghainese’s average income and two-thirds more of that in 2010. Thus, four categories were set as: ≤2308, (2308–4615], (4615–7692] and >7692. When annual income was analysed, students were excluded from the population in order to avoid possible bias.

Education was originally subdivided into 6 groups: below junior middle school, high school, technical/vocational school, junior college, college/university, and above university. Since most urban residents in China won’t go to work until he/she is above 18 years old and children are sent to school when they reach 6 years old. When they are 18 years old, they have received education with the time length of 12 years. We then dichotomized education by the length of the time they received education, less than 12 years and more than 12 years.

Housing condition manifested a remarkable change with economic development in China and indirectly reflected social-economic status [20,21]. Buildings built before 1980 are usually lower than three floors with old water pipes and sanitary facility. Water pipes of buildings built between 1980 and 2000 are of zincified steel. Pipes of buildings after 2000’s are of polyethylene. Drinking water in apartments lower than three floors is directly from water supply network, while that of higher floors comes from tank water. Therefore, housing condition described by building time and floors was categorized as follows: (1) before 1980’s (2) between 1980 and 2000 but live below 3rd floor (3) between 1980 and 2000 but live above 3rd floor (4) after 2000 but live below 3rd floor (5) after 2000 but live above 3rd floor and below half of the highest floor (6) after 2000 and above half of the highest floor.

Risk was perceived by subjective sensory feelings such as colour, smell, turbidity, taste and visible worm. Respondents were asked to recall any abnormality of aforementioned items in 2010.

Diarrhoea is the passage of 3 or more loose or liquid stools per day [22]. Respondents were asked to recall whether and how many times they suffered from drinking-water-related diarrhoea in 2010. In addition, those drinking barrelled and filtrated water were further inquired of their water hygiene habits, including frequency of barrelled water machine disinfection and filter replacement.

### Analyses

Descriptive and inferential statistics were undertaken to analyse the data. Chi-square test was performed to evaluate association between each study factor, between diarrhoea times and different water choices, as well as between diarrhoea times and water hygiene habits. When expected frequency was less than 5, Fisher’s exact test was used instead. The relation between each factor and different drinking water choices (tap water, barrelled/bottled water, filtrated water) was firstly assessed by univariable logistic regression. Four groups were set as follows: alternative water (barrelled/bottled water, filtrated water) vs tap water; barrelled/bottled water vs tap water; filtrated water vs tap water; and barrelled/bottled water vs filtrated water. Backward stepwise logistic regression model was used in each group. For each model, a receiver operating characteristic (ROC) curve was generated. The goodness of fit of the models was assessed by calculating the area under the curve (AUC) of the receiver operating characteristic (ROC) with 95% confidence intervals (95%CI) [23]. Agreement between domestic drinking water type and what one believed to be the cleanest was qualitatively assessed by the kappa statistic. Kappa values of 0.81–1 were interpreted as excellent agreement, 0.61–0.80 as good, 0.41–0.60 as moderate, 0.21–0.40 as slight, and 0–0.20 as poor [24]. Statistical analysis was performed with Stata/SE 11.0 (College Station, TX).

Historical data (1) percentage of domestic drinking water choices; (2) tap water satisfactory rate; (3) annual income and living years were available in 2001 [18]. Graphic method and chi-square test were also applied to reflect the change in drinking water choices.

## Results

### Socio-demographic characteristics of the sample

There were 199 (49.75%) male and 201 (50.25%) female, aged between 8 and 90 years with an average of 41.23 years. Constituent ratio of age was 9.75% for juveniles, 21.50% for young, 45.25% for the middle-aged, and 23.50% for the elder, respectively. Average annual income was $4879 and 32.50% of the respondents received education above junior college level. Percentages of those living in three different kinds of buildings were respectively 10.50%, 61.75% and 27.75%, in chronological order. Average living year in the present place was 18.36 years. Full-time students were especially listed given that they were economically dependent and their housing condition largely depended on their family. Details could be seen from Table 1.

**Table 1:** Socio-demographic characteristics of study population

**Characteristics**	**Study population**		**Excluded population**	**Shanghai population**	
	N (%)	Students (%)	N (%)	N (%)	Students
**Total**	400	44	16	13,793,900	1,962,900
**Gender**					
Male	199 (49.8)	23 (52.3)	8 (50)	(49.9)	
Female	201 (50.2)	21 (47.7)	8 (50)	(50.1)	
**Age (years)**					
<18	39 (9.8)	37 (84.1)	5 (31.3)	(10.4)	
18~	86 (21.5)	7 (15.9)	7 (43.7)	(24.1)	
35~	181 (45.3)	0	3 (18.8)	(43.0)	
60~	94 (23.5)	0	1 (6.3)	(22.5)	
**Education**					
≤12 years	270 (67.5)	39 (88.6)	9 (56.3)	(75.0)	
> 12 years	130 (32.5)	5 (11.4)	7 (43.8)	(25.0)	
**Annual Income ($)**					
≤2,308	51 (12.8)	44 (100)	6 (37.5)	----	
2,308.1 ~ 4,615	239 (59.8)	0	3 (18.8)	----	
4,615.1 ~ 7,692	68 (17)	0	5 (31.3)	----	
7,692.1~	42 (10.5)	0	2 (12.5)	----	
**Housing Condition**					
1	41 (10.3)	3 (6.8)	----	----	
2	106 (26.5)	10 (22.7)	----	----	
3	142 (35.5)	16 (36.4)	----	----	
4	24 (6)	5 (11.4)	----	----	
5	39 (9.8)	6 (13. 6)	----	----	
6	48 (12)	4 (9.1)	----	----	

The data of Shanghai population was gained from Shanghai Statistical Almanac 2010. Unavailable data were illustrated as dashed lines.

The average age of Shanghai residents was 36.64 years. Constituent ratios of the study in age and gender were similar to those of Shanghai. The annual income of Shanghai residents was $4898, which was also comparable. 25% of all respondents in Shanghai received higher education. The reason that ours were relatively high in both average age and percentage of higher education receivers was probably because we excluded populations below 7 years. In total, our sample was representative of that in Shanghai.

### Risk perception, belief and domestic drinking water choices of respondents

Respondents were aware of tap water quality from sensory property items of turbidity, colour, taste, smell and if worm was ever seen. According to their water consumption experience in 2010, 239 (59.75%) were satisfied with domestic tap water quality. In terms of turbidity, 57 (14.25%) thought tap water was slightly turbid, and 1 (0.25%) complained about severe turbidity. 30 (7.50%) reported abnormal colour, among which yellowish green was mostly reported. 38 (9.50%) respondents experienced abnormal smell, with 22 (5.50%) chlorine smell, 7 (1.75%) fish odour, 5 (1.25%) sulphur smell, and 4 (1%) others. 38 (9.50%) reported ever found worm in tap water. Taste was the more frequent given reason for the dissatisfaction of tap water. 106 (26.50%) respondents complained about the uncomfortable perception.

Respondents exhibited a different belief of what was the cleanest or safest water. 210 (52.50%) of the respondents thought tap water was the cleanest, sequentially came 77 (19.25%) filtrated water, 53 (13.25%) bottled water, 37 (9.25%) barrelled water and 23 (5.75%) respondents were “indifferent” towards the type of drinking water.

### Comparisons between 2001 and 2011

**Figure 1:**
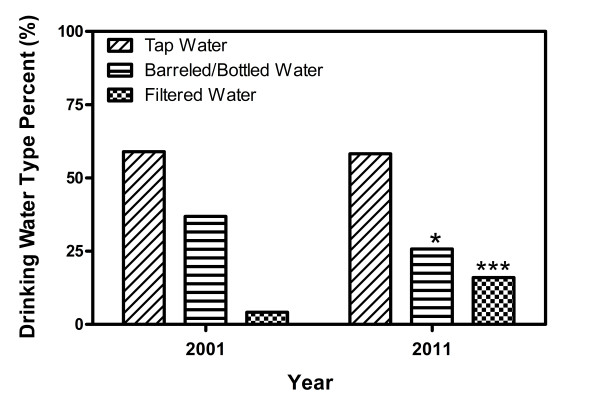
** Change in domestic drinking water choices during past decade.** The percentage of tap water use remained relatively stable, with only a 0.74% increase compared with that in 2011. A decrease in barrelled water by around 11.0% and an increase in filtrated water use by 11.85% were found. Information of domestic bottled water use wasn’t gathered in 2001, its percentage in 2011 was 3.25%. Statistical significance were found in difference of filtrated water (*P* < 0.001) and barrelled/bottled water (*P* < 0.05).

Changes occurred in personal income, living years, tap water satisfactory rate and domestic drinking water choices during the decade. Average annual income was $1218 in 2001, 1/4 of that in 2011. The average living years in present place was 15 years in 2001, while that of 2011 was 18 years, indicating a change in living place and housing condition among residents. Tap water satisfactory rate used to be 41.94% in 2001, 10.31% lower than that of 2011. The percentage of tap water use remained relatively stable, with only a 0.74% increase compared to that in 2011. Bottled water was not listed in 2001-study, therefore, we combined barrelled and bottled water together, and found a decrease by 11.11%. Percentage of household filtrated water use had increased by 11.85% (See Figure 1)

### Influential factors of domestic drinking water choices

Domestic drinking water choices were influenced by age, income, education, housing condition, taste, worm found and belief differences, whereas gender, transparency, smell, colour showed no relations in any regression model. A slight agreement (60.46%) was also found between what people recognized cleanest and what they actually drink at home, Kappa value = 0.36 (*P* < 0.001).

**Figure 2:**
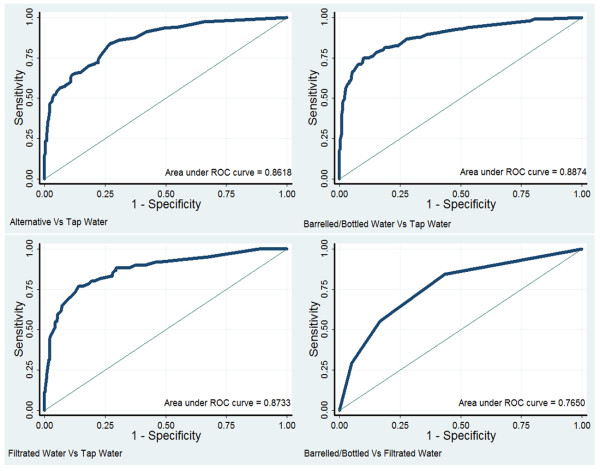
** Receiver operating characteristic (ROC) curve for four backward stepwise logistic regression models.** The areas under curve (AUC) and their 95% CI for each model were 0.86 (0.83–0.90), 0.89 (0.85–0.92), 0.87 (0.84–0.91) and 0.77 (0.70–0.83), respectively.

Backward stepwise logistic regression model was used to see how the influential factors affected domestic drinking water choices in each group. Respondents drinking tap water were regarded as reference in the first three groups, while filtrated water in the last. ROC curves of all four models showed good ability to distinguish different drinking water choices, with the AUC of 0.86 (0.83–0.90), 0.89 (0.85–0.92), 0.87 (0.84–0.91) and 0.77 (0.70–0.83), respectively (See Figure 2).

Belief was brought into the regression model in all groups. Education was included only in the group for alternative drinking water, indicating that respondents with higher education were inclined to choose alternative drinking water rather than tap water, but there existed no difference in the type of alternative drinking water. Income was included in groups for alternative water and bottled/barrelled water, while age was included in the model for bottled/barrelled water only. Housing condition was included in the group for filtrated water. Taste was included in the first three groups, indicating it a neglectable reason for abandonment tap water. Worm founded in tap water was also an influential factor in groups for alternate and filtrated water. Details could be seen from Table 2.

**Table 2:** Logistic regression models for different domestic drinking water choices

	**Alternate**		**Bottled/Barreled**		**Filtrated**		**Bottled/Barreled Vs (Filtrated)**	
	cOR (95% CI)	aOR (95% CI)	cOR (95% CI)	aOR (95% CI)	cOR (95% CI)	aOR (95% CI)	cOR (95% CI)	aOR (95% CI)
**Gender** (Male)	1.11 (0.91–1.35)	----	1.09 (0.87–1.38)	----	1.14 (0.87–1.51)	----	0.96 (0.70–1.31)	----
**Age** (18–35years)								
<18	1.11 (0.52–2.38)	----	1.02 (0.44–2.36)	0.97 (0.24–2.04)	1.36 (0.46–4.02)	----	0.75 (0.24–2.31)	----
35–59	0.62 (0.37–1.03)	----	0.49* (0.27–0.88)	0.45** (0.32–0.97)	0.95 (0.45–2.04)	----	0.51 (0.23–1.16)	----
≥60	0.47* (0.26–0.86)	----	0.33** (0.16–0.68)	0.36** (0.10–0.73)	0.83 (0.35–1.96)	----	0.4 (0.15–1.05)	----
**Education** (Lower level)	1.57* (1.03–2.40)	2.74** (1.38–5.44)	1.61 (0.99–2.62)	----	1.52 (0.85–2.71)	----	1.06 (0.56–2.01)	----
**Annual Income** (2308.1–4615)								
≤2,308	1.38 (0.30–6.33)	2.38 (0.86–6.67)	2.70 (0.58–12.54)	1.35 (0.83–23.51)	----	----	----	----
4,615.1–7,692	2.08** (1.20–3.58)	5.26** (1.61–16.67)	2.70** (1.44–5.07)	3.08** (1.26–6.39)	1.42 (0.67–2.99)	----	1.91 (0.84–4.31)	----
7,692.1-	1.25 (0.64–2.45)	3.57 (1.61–7.94)	1.87 (0.88–3.97)	1.14 (0.93–2.74)	0.60 (0.20–1.84)	----	3.10 (0.93–10.28)	----
**Housing Condition** (CAT 1)								
CAT 2	2.00 (0.86–4.62)	----	1.94 (0.72–5.21)	----	2.10 (0.56–7.89)	2.48 (0.50–12.28)	0.92 (0.20–4.31)	----
CAT 3	2.84** (1.27–6.38)	----	2.96* (1.15–7.61)	----	2.61 (0.72–9.43)	3.02 (0.43–21.16)	1.13 (0.26–5.01)	----
CAT 4	3.10* (1.04–9.23)	----	2.96 (0.84–10.50)	----	3.38 (0.66–17.25)	3.14 (0.67–14.85)	0.88 (0.137–5.576)	----
CAT 5	5.27** (1.99–13.97)	----	2.75 (0.82–9.27)	----	10.31** (2.61–40.82)	17.90** (3.39–94.42)	0.27 (0.05–1.36)	----
CAT 6	3.99** (1.57–10.10)	----	3.59* (1.21–10.63)	----	4.78* (1.18–19.31)	4.82* (1.02–21.27)	0.75 (0.15–3.72)	----
**Transparency** (Normal)	0.93 (0.55–1.57)	----	0.72 (0.49–1.06)	----	1.18 (0.83–1.68)	----	0.61* (0.39–0.97)	----
**Colour** (Normal)	0.92 (0.59–1.44)	----	0.58 (0.31–1.08)	----	1.27 (0.82–1.97)	----	0.46* (0.23–0.91)	----
**Taste** (Good)	3.50*** (2.65–4.63)	5.48*** (3.50–8.56)	4.04*** (2.98–5.49)	6.44*** (3.91–10.60)	2.78*** (1.97–3.91)	5.04*** (2.74–9.26)	1.46* (1.06–2.00)	----
**Smell** (Normal)	1.01 (0.72–1.41)	----	0.84 (0.54–1.30)	----	1.25 (0.83–1.90)	----	0.67 (0.40–1.12)	----
**Worm founded** (Never)	1.14 (0.58–2.24)	2.95* (1.28–6.81)	0.59 (0.21–1.61)	----	2.33* (1.08–5.04)	3.35* (1.22–9.15)	0.22** (0.07–0.66)	----
**Belief** (Tap water)								
Barrelled water	19.49*** (7.65–49.66)	8.62 *** (3.51–21.20)	27.67*** (10.50–72.87)	9.68*** (2.91–32.16)	5.19* (1.18–22.74)	5.14 (0.92–28.86)	5.33* (1.40–20.36)	5.61* (1.47–21.38)
Bottled water	5.75*** (3.02–10.94)	2.39** (1.32–4.23)	7.06*** (3.49–14.28)	3.61** (1.51–8.60)	3.46* (1.28–9.38)	2.59 (0.83–8.05)	2.04 (0.72–5.77)	2.55 (0.88–7.39)
Filtrated water	6.60*** (3.73–11.69)	3.04*** (1.81–5.10)	3.18** (1.51–6.68)	1.66 (0.64–4.31)	12.60*** (6.15–25.79)	11.54*** (5.05–26.39)	0.25** (0.11–0.60)	0.51** (0.11–0.56)

* *P* -value < 0.05; ** *P* -value < 0.01; *** *P* -value < 0.001.

Crude and adjusted odds ratios and their 95% CI were shown above.

Tap water choice was the control in the first three models, while filtrated water was the control in the last model.

Housing condition was divided into 6 categories, category 1 (CAT 1) was apartments built before 1980’s.

Transparency, colour, taste, smell and worm ever founded were five aspects considering respondents’ household tap water.

Variables excluded from the models were illustrated as dashed lines.

### Drinking water choices, diarrhoea frequency and water hygiene habits

Percentage of domestic drinking water choices was as follows: 58.25% using tap water, 22.50% barrelled water, 3.25% bottled water and 16.00% filtrated water. 171 (42.75%) respondents reported suffered from drinking-water-related diarrhoea. 149 (87.13%) of them experienced less than 5 times. No statistical significance was found between diarrhoea and drinking water choices. However, those taking alternate drinking water showed a higher diarrhoea rate.

For those drinking barrelled water (n = 90), 21 (23.33%) disinfected their barrelled water machine every month, 20 (22.22%) every 3 months, 21 (23.33%) every half year and 28 (31.11%) more than one years. A total of 37 (41.11%) reported suffering from drinking-water-related diarrhoea. For those drinking filtrated water (n = 64), 10 (15.63%) replaced their filters at least once every three months, 28 (43.75%) replaced in an annual base, 26 (40.63%) replaced less frequent or never replaced since equipped. 30 (46.88%) of them reported suffering from diarrhoea.

## Discussion

Drinking water choices reflect not only the history and development of a society, an economy and a culture, but also awareness and concept of public health and drinking water hygiene. Past three decades witnessed a rapid economic growth, introduction of new technologies and increase international exchanges in China. Reform from planned economy to the market one has greatly improved life of Chinese people and influenced their lifestyle and health concept [25]. However, few researches have ever investigated change and the possible influential factors on drinking water choices and water consumption in China. We conducted a cross-sectional survey in Shanghai, a metropolitan fully representing the economic and social development of China, to investigate change in domestic drinking water choices and water consumption and its potential health issues in urban residents. Domestic drinking water choices diversified in China, with tap water a mainstream but alternative drinking water noticeably changed. Domestic filtrated water increased and barrelled/bottled water decreased, compared with that decade ago. Domestic drinking water choices were correlated with age, education, annual income, housing condition, risk perception and belief in what kind of drinking water the cleanest. Strong correlation was found between one’s belief in what type of water the cleanest and one’s domestic drinking water choices (*P* < 0.001) by using backward stepwise logistic regression model. However, misunderstanding proved existed from rates of self-report drinking-water-related diarrhoea, filter replacement and barrelled water machine disinfection. Therefore, adverse effects and potential health risks induced by change in drinking water choices can’t be ignored.

The development of economy provides diversified drinking water choices. Our study showed that 58.25% of the residents mainly drank boiled tap water. However, the percentage of domestic filtrated and barrelled/bottled water use had surpassed 40%, with an increase by 11.85% in filtrated water (*P* < 0.001) and a decrease by 11.11% in barrelled/bottled water (*P* < 0.05), compared with that ten years ago. The reason that domestic filtrated water rose in use was attributed to many factors, but the development of economy, the improvement of residents’ living conditions and marketing effect were among the very important. Although barrelled/bottled water was much easier to access than tap water, recent negative reports for barrelled/bottled water from home and aboard led to its decrease in household use [26-31].

Our findings that differences in age, education, income and personal perception affected drinking water choice were similar with what found in western countries [5-8,10]. However, these influential factors showed characteristics rooted from Chinese tradition and economic background. 49.23% of the higher educated respondents chose alternative water, while only 38.15% lower educated did so. As shown in Additional file 2: Table S1, annual income interacted with education (*P* < 0.001). 65.60% of the higher educated respondents owned a higher annual income (>$4615), while that for lower educated was only 12.12%. No wonder the higher income selected more alternative and barrelled/bottled water than the lower income (48.18% vs 39.31% and 33.64% vs 22.76%).

We also found the middle-aged and the elder (≥35 years old) chose less barrelled/bottled water at home than the juveniles and the young (24.78% vs 43.40%). This was because of the influence by traditional thrifty life philosophy and the long-term propaganda for using boiled tap water. On the other hand, it may be attributed to China’s family planning policy carried out 30 years ago. More than 90% of the respondents under 35 years old were the only child in the family, who received much more care and resources than their parents [32,33].

We were the first to discover that housing condition influenced residents’ drinking water choices and water consumption behaviours. In China, housing conditions was a comprehensive embodiment of one’s and his/her family’s income level, social status, educational and cultural background. Respondents living in buildings built after 2000 were much more likely to choose filtrated water than others (cOR = 1.19 95%CI 1.07–1.32). The reasons lay in two aspects: (1) Centralized filtrated water was usually supplied in communities built after year 2000 due to improvement of residential environment. Even if without centralized filtrated water, many residents would equipped a filter at their water inlets. (2) Respondents living in buildings built after 2000 enjoyed a higher annual income and education, with 37.94% belonging to higher income and 51.35% higher education, compared to 23.88% and 25.26% for those living elsewhere.

Knowledge-attitude-practice (KAP) model assumed that knowledge, attitude and practice inter-related with one another [34]. Personal health belief affected one’s behaviour [35]. We found respondents’ drinking water choices (practice) correlated with their belief in what type of water the cleanest (attitude) by using backward stepwise logistic regression model. However, looking further, we found respondents’ belief was subjective, even self-contradictory and vague in logic. For example, the respondent could hardly tell why choose a certain type of water. Moreover, 14.06% of the respondents expressed bad smell and taste and even found bloodworm (midge larvae) in their tap water, but still regarded tap water was a prior choice. These were in line with what Ward discovered in their research that there existed confusion about what kind of drinking water the cleanest in the general public [8].

**Figure 3:**
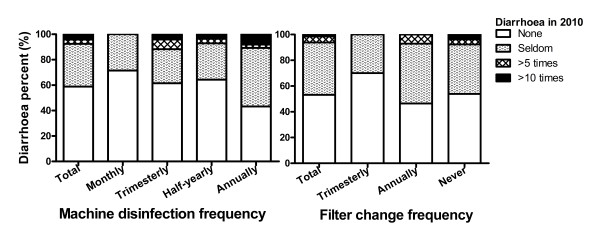
** Diarrhoea times among different frequency of barrelled water machine disinfection and filter replacement.** Self-report diarrhoea in 2010 was compared among barrelled and filtrated water users. 41.11% of barrelled water users and 46.88% of filtrated water users reported diarrhoea in 2010. The more often the frequency of disinfection and filter replacement, the less chance and severity of self-report diarrhoea

Bacteria qualified rate reached 100% in finished water of water plant and pipe net water at national monitoring points for water quality [36]. Barrelled/bottled water was also tested the quality before selling. However, self-report drinking-water-related diarrhoea in 2010 existed in all kinds of water. The diarrhoea rates for filtrated, barrelled/bottled and tap water were 46.88%, 44.66%, 40.77%, respectively, though there was no statistical significance (See Table 3). It was astonishing that respondents drinking alternative water (barrelled/bottled & filtrated water) suffered more. A tendency was found that the higher the frequency for filter replacement and barrelled machine disinfection, the lower the chance and severity of diarrhoea, although without statistical significance (See Figure 3). The frequency for replacement or disinfection was usually judged by time instead of water volume. Some respondents even didn’t know when and how to replace filter or disinfect machine. They either never did so or did in an irregular base. We thought it may be the effectiveness of drinking water sanitary treatment rather than drinking water type that influence the difference of diarrhoea rate.

**Table 3:** Self-report diarrhoea in 2010

	**Diarrhoea times and percentages**			
	**< 5 times**	**< 10 times**	**More**	**Total**
	**n (%)**	**n (%)**	**n (%)**	**n (%)**
**Tap Water**	86 (36.91)	4 (1.72)	5 (2.15)	95(40.77)
**Bottled/Barrelled Water**	37 (35.92)	4 (3.88)	5 (4.85)	46(44.66)
**Filtrated Water**	30 (46.88)	2 (3.13)	0	32(50)

Pearson *X*^2^ = 7.76,*P* >0.05.

This was the first research to investigate change in domestic drinking water choices and water consumption behaviours among urban residents in China. Diversification of drinking water choices was inevitable under the background of market economy. The increase in household filtrated water and the decrease in barrelled water reflected the outcome of drinking water market. Although the influential factors of drinking water choices were similar with those found in developed countries, reasons lay behind were of Chinese characteristics rooted in Chinese unique culture, society and economy. All types of water could induce diarrhoea, indicating a necessity to further strengthen propaganda and health education and to instruct residents in effective disinfection and sanitary methods according to different drinking water choices. Government and centres for disease control should be urged to enhance management and supervision of drinking water quality so as to protect public health.

Selection bias may have occurred in that respondents with both time and willingness were recruited, but as questionnaires were done in different days and different locations in Yangpu district, we believe such bias were decreased to a large extent. Given that respondents were asked to recall some of their water consumption experience in 2010, the potential for recall bias was possible. However, we believe it was attenuated as the influential factors and water consumption habits that we focused were relatively stable. Though misclassification of drinking-water related diarrhoea may have occurred, it was still possible to infer related diarrhoea could happen to residents drinking any type of water. We think it would be better if we had asked those respondents the type of water they used to drink and the reason for change.

## Conclusions

We found consumption of tap water remained a majority, while the use of alternative drinking water changed, with an increase in filtrated water and decrease in bottled/barrelled water. Many factors including personal health belief, housing condition, risk perception affected domestic drinking water choice in urban China. However, reasons for these influential factors were rooted in Chinese traditional culture, special social structure and stage of economic development. All types of water could induce diarrhoea and misunderstanding of water hygiene accounted for high proportion. Administrative department should provide related health education, knowledge and effective measures to safeguard public health in accordance with various drinking water choices.

## Competing interests

The authors declare that they have no competing interests.

## Authors’ contributions

WQ and HC conceived and designed the whole study. YZ, LM, FL and QS were involved with the study design, respondents recruitment, and data collection. HZ, XW, DT and GH were involved with the modification of the questionnaire. HC performed statistical analysis and drafted the manuscript. WQ and WZ provided critical input into the manuscript. All authors read and approved the final version.

## Pre-publication history

The pre-publication history for this paper can be accessed here:

http://www.biomedcentral.com/1471-2458/12/450/prepub

## Supplementary Material

Additional file 1 Questionnaire for Water Consumption Habits. Click here for file

Additional file 2 **Table S1.** Interaction among studied influential factors. Click here for file
