# Supplementary material for: Change of water consumption and its potential influential factors in Shanghai: A cross-sectional study
Source: BMC Public Health. 2012 Jun 18;12:450. doi: 10.1186/1471-2458-12-450 (PMC3539858; doi:10.1186/1471-2458-12-450)
Supplement: Additional file 1 — Questionnaire for Water Consumption Habits. [file 1471-2458-12-450-S1.doc]

## Supplementary Material, Table 1 - Interaction among studied influential factors

|  | **Gender** | **Income** | **Education** | **Age** | **Housing Index** | **Transparency** | **Smell** | **Color** | **Taste** | **Worm founded** | **Belief** |
| --- | --- | --- | --- | --- | --- | --- | --- | --- | --- | --- | --- |
| **Gender** |  |  |  |  |  |  |  |  |  |  |  |
| **Income** | 29.46  (0.000)** |  |  |  |  |  |  |  |  |  |  |
| **Education** | 12.08  (0.034)* | 190.35  (<0.001)** |  |  |  |  |  |  |  |  |  |
| **Age** | 0.50  (0.920) | 148.93  (<0.001)** | 259.74  (<0.001)** |  |  |  |  |  |  |  |  |
| **Housing Index** | 4.87  (0.088) | 27.50  (0.025)* | 32.14  (<0.001)** | 18.73  (0.226) |  |  |  |  |  |  |  |
| **Transparency** | Fisher’s  (0.478) | 7.85  (0.049)* | Fisher’s  (0.173) | 3.28  (0.350) | 8.99  (0.109) |  |  |  |  |  |  |
| **Smell** | Fisher’s  (1.000) | 1.35  (0.716) | Fisher’s  (0.469) | 4.95  (0.176) | 3.72  (0.591) | Fisher’s  (<0.001)*** |  |  |  |  |  |
| **Color** | Fisher’s  (0.343) | 7.43  (0.059) | Fisher’s  (0.026)* | 7.98  (0.046)* | 5.30  (0.381) | Fisher’s  (<0.001)*** | Fisher’s  (<0.001)*** |  |  |  |  |
| **Taste** | Fisher’s  (1.000) | 0.363  (0.948) | Fisher’s  (0.468) | 1.98  (0.578) | 3.86  (0.570) | Fisher’s  (0.422) | Fisher’s  (<0.001)*** | Fisher’s  (0.393) |  |  |  |
| **Worm founded** | Fisher’s  (0.731) | 5.56  (0.135) | Fisher’s  (0.059) | 7.51  (0.057) | 11.13  (0.049)* | Fisher’s  (<0.001)*** | Fisher’s  (0.006)** | Fisher’s  (<0.001)*** | Fisher’s  (0.329) |  |  |
| **Belief** | 4.95  (0.293) | 20.19  (0.017)* | 5.83  (0.12) | 20.35  (0.016)* | 20.64  (0.149) | 2.10  (0.551) | 4.41  (0.220) | 0.291  (0.962) | 47.36  (<0.001)*** | 0.62  (0.891) |  |

Pearson chi-square or Fisher’s exact with its p-value are shown above

* p-value<0.05; ** p-value<0.01; ***p-value<0.001
