# Supplementary material for: Change of water consumption and its potential influential factors in Shanghai: A cross-sectional study
Source: BMC Public Health. 2012 Jun 18;12:450. doi: 10.1186/1471-2458-12-450 (PMC3539858; doi:10.1186/1471-2458-12-450)
Supplement: Additional file 2 — Table S1. Interaction among studied influential factors. [file 1471-2458-12-450-S2.pdf]

Please select **one best answer** for each question. Two more choices is regarded proper only for questions we specially marked out.

Questionnaire for Water Consumption Habits

I. Individual Information

1. **Gender**: (1) Male (2) Female
2. **Place of birth:** ________________
3. **Date of birth**: __________________
4. **Home address:** ______Room ______Building _________Road _______Lane
5. **How long have you lived in this place?** _________years
6. **Education**: (1) Junior middle school or below (2) High school (3) Technical school
   (4) Junior college (5) College/university (6) Above university
7. **Current occupation:** _____________
   7.1. **Previous occupation:** _______________
8. **Your annual income in 2010:** (1) $ 0-2,308 (2) - $4,615 (3) - $7,692 (4) > $7,692
9. **Do you expose to water in your current/previous occupation?**

If not, please go to II. Habits of Drinking Water.

9.1. **Season of exposure:** (1) Spring (2) Summer (3) Autumn (4) Winter
(you can choose more than one option)

9.2. **Frequency of exposure:** (1) 1-2 times per month (2) 1-2 times per week
(3) 3-4 times per week (4) ≥5 times per week

9.3. **Time length of exposure:** (1) 0-1 hour (2) - 2 hours (3) - 4 hours (4) - 6 hours (5) > 6 hours

9.4. **Type of water:** (1) Tap water (2) Boiled tap water (3) Others ___________

9.5. **Protection measure:** (1) No protection (2) Gloves only (3) Mask only
(4) Gloves and mask (5) Others__________________

II. Habits of Drinking Water

1. **What kind of water do you usually take at home?**
   (1) Tap water (2) Barreled water (3) Bottled water (4) Filtrated water
2. **What kind of water do you usually take at work?**
   (2) Tap water (2) Barreled water (3) Bottled water (4) Filtrated water
3. **In spring/autumn, how much water do you drink on average?**
   ________ cup (______ml/cup)
4. **In summer, how much water do you drink on average?**
   ________ cup (______ml/cup)
5. **In winter, how much water do you drink on average?**

________ cup (______ml/cup)

6. Do you ever process your drinking water using following methods?

(1) Heated by microwave oven (2) Frozen by fridge (3) Neither (4) Both

7. What kind of water do you usually use for tea, coffee or soybean?

(1) Boiled tap water (2) Boiled barreled water (3) Both (4) Others _________________

8. Do you drink overnight water?

(1) Never (2) 1-2 times per month (3) 1-2 times per fortnight (4) ≥3 times per week

9. Do you drink tap water directly? (1) Never (2) Occasionally (3) Often (≥ once a week)

10. What is your kettle made of? (1) Aluminum (2) Stainless steel (3) Plastic (4) Others

11. How long will you continue to heat your kettle after the water is boiled?

(1) ≤1 min (2) -5 min (3) -10 min (4) > 10 min

12. Do you drink tea?

If not, please go to Q 13.

12.1 **Which kind of tea do you usually drink?** (1) Green tea (2) Black tea (3) Oolong tea (4) Others __________

12.2 **How much do you drink daily?** ________ cup (______ml/cup)

12.3 **Do you drink overnight tea?** (1) Never (2) 1-2 times per month (3) 1-2 times per week (4) ≥3 times per week

13. Do you drink soybean, milk or juice?

(1) Never (2) 1-2 times per week (3) 3-5 times per week (3) 6-7 times per week (4) More than once per day

14. Do you drink beverage brewed by milk powder, wheat or etc.?

If not, please go to Q 15.

14. 1 **Frequency:** (1) 1-2 times per week (2) 3-5 times per week (3) 6-7 times per week (4) More than once per day

14.2 **Volume for each time:** ____________ml

15. Do you drink coffee?

If not, please go to Q 16.

15.1. **Drink frequency:** (1) 1-2 times per month (2) 1-2 times per week (3) 3-5 times per week (4) > 5 times per week

15.2. **Drink volume for each time:** ____________ml

16. Do you prefer hot or cold drink?

(1) Hot drink (2) Cold drink (3) Hot for winter, others cold (4) Cold for summer, others hot (5) Don't mind

17. What is your drinking-water bottle made of? (1) Plastic (2) Non plastic

18. How often do you clean your drinking water container?

(1) Once every day (2) 2-3 times a week (3) Once a week (4) More than once a week

III. Bathing Habits

1. **Your usual way of washing yourself is:** (1) Shower (2) Bath (3) Rubbing with towel
2. **In spring/autumn, your frequency of shower is:** (1) Once every day (2) Once every 2 days
   (3) Once every 3-4 days (4) Once every 5-7 days (5) Longer
   2.1 **Time length of each shower:** (1) In 10 min (2) -20 min (3) -30 min (4) -45 min
   (5) -60 min (6) > 60 min
3. **In summer, your frequency of shower is:** (1) More than once every day (2) Once every day
   (3) Once every 2 days (4) Once every 3 days (5) Longer
   3.1 **Time length of each shower:** (1) In 10 min (2) -20 min (3) -30 min (4) -45 min
   (5) -60 min (6) > 60 min
4. **In winter, your frequency of shower:** (1) Once every day (2) Once every 2 days (3) Once every 3-4 days
   (4) Once every 5-7 days (5) Longer
   4.1 **Time length of each shower:** (1) In 10 min (2) -20 min (3) -30 min (4) -45 min
   (5) -60 min (6) > 60 min
5. **Do you bath in bathtub?**
   If not, please go to Q6.
   5.1 **Season to bath:** (1) Spring (2) Summer (3) Autumn (4) Winter **[You may choose one more choice]**
   5.2 **Frequency of bath:** (1) Daily (2) Once every 2-4 days (3) Once every 5-7 days (4) Longer
   5.3 **Time length of each bath:** (1) In 15 min (2) -30 min (3) -45 min (4) -60 min (5) > 60 min
6. **Do you bath with cold water?**
   If not, please go to Q7.
   6.1 **Season to bath with cold water:** (1) Spring (2) Summer (3) Autumn (4) Winter
   [You may choose one more choice]
7. **Did you ever bath at public bathing place in 2010?**
   If not, please go to Q8.
   7.1 **Frequency of bath:** (1) <10 times (2) 10-50 times (3) >50 times
   7.2 **Time length of each bath:** (1) In 15 min (2) -30 min (3) -60 min (4) > 60 min
8. **Do you sauna?**
   If not, please go to Q9.
   8.1 **Sauna type:** (1) Dry sauna (2) Steamed sauna (3) Both
   8.2 **When to sauna:** (1) Spring (2) Summer (3) Autumn (4) Winter **[You may choose one more choice]**
   8.3 **Frequency:** (1) Once every day (2) Once every 2-4 days (3)Once every 5-7 days
   (4) Once every 2 weeks (5) Once every month
   8.4 **Time length of each sauna:** (1) In 5 min (2) -10 min (3) -20 min (4) -30 min (5) > 30 min
9. **Frequency of rubbing yourself with towel:** (1) Never (2) Rubdown whenever fail to shower/bath
   (3) Others ________
   9.1 **Water temperature:** (1) Hot water (2) Cold water (3) Hot for winter, others cold

(4) Cold for summer, others hot

10. Do you take foot bath? (1) Never (2) Yes, for 4 seasons (3) Yes, only for winter

11. You wash face with: (1)Cold water (2)Hot water (3)Hot for winter, others cold

(4) Cold for summer, others hot

IV. Other Habits

1. Do you wash dishes at home?

If not, please go to Q2.

1.1 **Frequency of washing:** (1) Once every day (2) Once every 2 days

(3) Once every 3-5 days (4) Once every 6-7 days

1.2 **Time length of each wash:** (1) In 10 min (2) -30 min (3) >30 min

1.3 **Protection measure** (1) With gloves every time (2) Gloves only for winter (3) Never

2. Do you wash clothes by hands at home?

If not, please go to Q3.

	In sping/autumn	In summer	In winter
**Frequency of washing:**	(1) Daily	(1) Daily	(1) Daily
	(2) 2-5 times per week	(2) 2-5 times per week	(2) 2-5 times per week
	(3) 6-7 times per week	(3) 6-7 times per week	(3) 6-7 times per week
	(4) Never	(4) Never	(4) Never
**Time length of each wash:**	(1) In 10 min	(1) In 10 min	(1) In 10 min
	(2) -30 min	(2) -30 min	(2) -30 min
	(3) -60 min	(3) -60 min	(3) -60 min
	(4) > 60 min	(4) > 60 min	(4) > 60 min

2. 1 **Protection measure:** (1) With gloves every time (2) Gloves only for winter (3) Never

3. Do you wash clothes by washing machine at home?

If not, please go to Q4.

3. 1 **How many times do you wash clothes for more than 1 hour every month?** _________times

4. Do you iron your clothes?

If not, please go to Q5.

4.1 **Frequency of iron:** (1) Daily (2) Once every week (3) Once every 2 weeks

(4) Once every month (5) Others______

4.2 **Time length of each ironing:** (1) In 10 min (2) -30 min (3) -60 min (4) -120 min

4.3 **Temperature of iron:** (1) Low temp (2) High temp (3) Both

5. Do you steam-bathing your face?

If not, please go to Q6.

5.1 **Frequency of steam:** (1) Daily (2) Once every 2-4 days (3) Once every 5-7 days

(4) Once every 2 weeks (5) Once every month

5.2 **Time length of each steam-bathing:** (1) In 10 min (2) 10-30 min (3) 30-60 min (4) > 60 min

5.3 **How long have you kept this habit? __________**years

6. Do you swim?

If not, please go to Q7.

6.1 **How many months do you swim in a year (at least twice a month)? ________months**

6.2 **How many times do you swim in these months?** (1) < 5 times (2) 5-10 times

(3) 11-15 times (4)16-20 times (5) ≥ 20 times

6.3 **Where do you swim?** (1) Indoor swimming pool (2) Outdoor swimming pool

(3) River (4) Sea water

6.4 **Time length of each swimming:** (1) In 30 min (2) -60 min (3) - 90 min (4) -120 min

(5) > 120 min

6.5 **How long have you kept this habit? ________**years

7. How long do you sit every day? (1) ≤ 8 hours (2) -10 hours (3) -12 hours (4) > 12 hours

8. Do you smoke now? (1) No (2) Occasionally (3) Often (4) Everyday

9. Do you drink alcohol? (1) Never (2) Occasionally (3) Often (4) Everyday

V. Health Status

1. Weight: _________ (kg) **Height: _________** (cm)

2. Do you have diabetes? (1) No/don't know (2) ≤ 5 years (3) ≤ 10 years (4) More than 10 years

3. Do you have constipation? (1) Never (2) Occasionally (3) Often

4. Do you have enteritis? (1) Never (2) Acute enteritis

(3) Chronic enteritis (duration > 2 months) (4) Not sure

5. Did you suffer from drinking-water related diarrhea in 2010?

(1) None (2) < 5 times (3) 5- 10 times (4) > 10 times

6. How many times were you diagnosed of drinking-water related diarrhea by doctor in 2010?

_____________times

7. Do you think above-mentioned diarrhea was associated with drinking water habits?

(1) Yes, they greatly associated. (2) Yes, but have small association. (3) I don't think so.

VI. Drinking Habits for Your Family

1. Does your family drink barreled water ?

If not, please go to Q2.

1.1 **How often do you disinfect your barrelled water machine?**

(1) Once a month (2) Once a trimester (3) Once half year (4) Once a year

(5) More than one year (6) Never

1.2 **How do you disinfect your barreled water machine? (you may choose more than one option)**

(1) Water (2) Vinegar (3) Ethanol (4) Chemical reagent (5) Professional disinfection

2. Does your family drink filtrated water?

If not, please go to Q3.

2.1 **How often do you replace your filter?**

(1) Once a month (2) Once a trimester (3) Once half year

(4) Once a year (5) More than one year (6) Never

2.1 **What's your filter made of? __________**

3. What does your family usually do with tap water/boiled tap water?

(1) Drinking (2) Brushing teeth (3) Washing face and body (4) Cooking

(5) Washing vegetables (6) Washing fruits

4. Does your family use vacuum bottle to store boiled water?

If not, please go to Q5.

4.1 **How often do you clean the bottle?**

(1) More than once a week (2) Once a week (3) Once every fortnight (4) Once a month (5) Longer

5. Quality of your domestic tap water

5.1 **Turbidity**

(1) Transparent (2) Slightly turbid (3) Moderately turbid (4) Severely turbid

5.2 **Color**

(1) Colorless (2) Others_________

5.3 **Taste**

(1) Good (2) Just so so (3) Uncomfortable/Bad

5.4 **Smell**

(1) Normal (2) Abnormal ____________

6. Did you ever find bloodworm in the water in 2010?

(1) Never (2) 1-5 times (3) 6-10 times (4) > 10 times

7. How does your family treat retained water? (Retained water is tap water stayed in the pipe for more than 6 hours.)

(1) Boiled for drinking (2) Brushing teeth (3) Washing face and body

(4) Cooking (5) Cleaning (6) Discarded

8. Do you have child? (If having one more child, just provide the following information for the first child)

If not, please go to VII. Subjective Questions.

8.1 **Was your child born in Shanghai?** (1) Yes (2) No

8.2 **How old is he/she?** _______

8.3 **Was he/she breast-fed?** (1) Yes, for ______months (2) No

8.4 **Was he/she fed with milk substituents during lactation period?**

(1) No (2) Yes

a. **Type of substituents:** (1) milk powder (2) milk (3) others___________

b. **Daily drink volume : ___________ For how long: __________** months

8.5 **Did he/she have baby-swimming before 3 years old?**

(1) No (2) Yes

a. **Frequency of swimming:** (1) 1-3 times (2) 4-10 times (3) > 10 times

b. **Time length of each swimming:** (1) 15-20 min (2) > 20 min

8.6 **Did he/she have drinking water related diarrhea before 3 years old?**

(1) Never (2) Seldom (3) Occasionally (4) Often (5) Not for sure

VII. Subjective Questions

1. What kind of water do you think is the cleanest/safest for drinking?

(1) Boiled water (2) Bottled Water (3) Barrelled Water (4) Filtrated Water (5) Don't mind

2. Do you know the reason why we need to chlorinate our water?

(1) To kill bacteria (2) To remove organic pollutants (3) I don't know

3. Have you ever heard of disinfection by-products?

(1) No, I've never heard of (2) Yes, it is good for my health (3) Yes, it is bad for my health

4. Please list ways of disinfection in water treatment process you know:

____
